# *CELSR3* is a prognostic marker in HNSCC and correlates with immune cell infiltration in the tumor microenvironment

**DOI:** 10.1007/s00405-024-08566-4

**Published:** 2024-03-20

**Authors:** Zhongbiao Wu, Zhongyan Zhu, Weikun Wu, Shiping Hu, Jian Cao, Xinmei Huang, Qiang Xie, Chengcheng Deng

**Affiliations:** 1 Department of Otolaryngology, Jiangxi Hospital of Integrated Traditional Chinese and Western Medicine, 90 Bayi Avenue, Xihu District, Nanchang, 330003 Jiangxi China; 2 Department of Rehabilitation, Jiangxi Hospital of Integrated Traditional Chinese and Western Medicine, Nanchang, 330003 China; 3 Department of Otolaryngology, Affiliated Hospital of Jiangxi University of Traditional Chinese Medicine, Nanchang, 330019 China

**Keywords:** Head and neck squamous cell carcinoma (HNSCC), *CELSR* receptor 3 (*CELSR3*), Prognosis, Tumor microenvironment, Tumor immune cell infiltration

## Abstract

**Purpose:**

To look at the diagnostic value of the *CELSR* receptor 3 (*CELSR3*) gene in head and neck squamous cell carcinoma (HNSCC) and its effect on tumor immune invasion, which is important for enhancing HNSCC treatment.

**Methods:**

Several bioinformatics tools were employed to investigate *CELSR3*’s putative oncogenic pathway in HNSCC, and datasets from The Tumor Genome Atlas (TCGA), Tumor Immune Estimation Resource (TIMER), Gene Expression Profile Interaction Analysis (GEPIA) and LinkedOmics were extracted and evaluated. *CELSR3* has been linked to tumor immune cell infiltration, immunological checkpoints, and immune-related genes. *CELSR3*’s putative roles were investigated using Gene ontology (GO), Kyoto Encyclopedia of Genes and Genomes (KEGG), and pathway enrichment analysis. The expression level of *CELSR3* in HNSCC tissues and cells was detected by RT-qPCR. The effects of *CELSR3* on proliferation of HNSCC cells were detected by CCK-8 assay.

**Results:**

*CELSR3* was shown to be expressed differently in different types of cancer and normal tissues. *CELSR3* gene expression was linked to pN-stage and pM-stage. Patients with high *CELSR3* expression also have a well prognosis. *CELSR3* expression was found to be an independent predictive factor for HNSCC in both univariate and multivariate Cox regression analyses. We discovered the functional network of *CELSR3* in HNSCC using GO and KEGG analysis. *CELSR3* expression levels were found to be favorably associated with immune cell infiltration levels. Furthermore, *CELSR3* expression levels were significantly correlated with the expression levels of many immune molecules, such as MHC genes, immune activation genes, chemokine receptors, and chemokines. *CELSR3* is highly expressed in HNSCC tissues and cells. *CELSR3* overexpression significantly inhibited the proliferation of HNSCC cells. *CELSR3* expression may affect the immune microenvironment and, as a result, the prognosis of HNSCC.

**Conclusion:**

*CELSR3* expression is elevated in HNSCC tumor tissues, and high *CELSR3* expression is associated with well prognosis, which inhibited the proliferation of NHSCC cells. *CELSR3* has the potential to influence tumor formation by controlling tumor-infiltrating cells in the tumor microenvironment (TME). As a result, *CELSR3* may have diagnostic significance in HNSCC.

## Introduction

Head and neck squamous cell carcinomas (HNSCCs) are the most prevalent malignancies of the head and neck and emerge from the mucosal epithelium in the oral cavity, pharynx, and larynx. HNSCC is the sixth most frequent cancer in the world, accounting for 890,000 new cases and 450,000 deaths in 2018 [11]. Although tumor excision combined with chemotherapy and radiotherapy has improved patients’ prognoses to some extent, advanced cancer is still prone to spread and worsening. As a result, it is critical to investigate more effective treatment strategies for HNSCC patients. Such as targeted therapy. However, in typical studies of targeted therapy for malignant tumors, the molecular pathways appear to be too complex for researchers to fully comprehend. So far, no useful biomarkers have been discovered.

CThe Cadherin EGF LAG seven-pass G-type (*CELSR*) family is derived from the Cadherin EGF LAG seven-pass G-type receptor and is categorized as a specific subgroup of adhesion G-protein-coupled receptor due to Cadherin repeating at the far N-terminal [29]. *CELSR* receptor 3 (*CELSR3*) has nine cadherin domains that operate as homophilic binding areas and seven EGF-like domains that are involved in receptor-ligand interactions and cell adhesion. It is thought to play an important role in cell–cell contacts [14, 24]. *CELSR3* has been linked to a variety of cancers, including hepatic, prostate, and lung adenocarcinomas [4, 17, 20]. However, it has not been determined whether *CELSR3* impacts the prognosis of HNSCC.

The Tumor Genome Atlas (TCGA), Tumor Immune Estimation Resource (TIMER), Gene Expression Profile Interaction Analysis (GEPIA), and LinkedOmics datasets were extracted and analyzed in this study to explore the potential oncogenic mechanism of *CELSR3* in HNSCC. The correlation between *CELSR3* and tumor immune cell infiltration, immune checkpoint, immune-related genes, and immune checkpoint blockade (ICB) was detected. Gene ontology (GO) and Kyoto Encyclopedia of Genes and Genomes (KEGG), pathway enrichment analyses were applied to investigate the potential functions of *CELSR3*. These findings offer fresh perspectives on *CELSR3*'s role as well as brand-new targets for HNSCC diagnosis and prognosis.

Immunotherapy has been used to treat HNSCC patients in recent years, transforming how HNSCC is treated [27]. It is widely recognized that tumor-infiltrating immune cells (TIICs) have an impact on the immune system, deal with abnormal biological activities in a complex manner, and are crucial in the body’s immune response to immunotherapy [28]. Furthermore, genes associated with immunological components can influence immune function [12]. Furthermore, cytokine and immune checkpoint blockade therapy have become treatment options for a variety of cancers, but no research has been conducted to determine whether *CELSR3* overexpression influences the tumor immune microenvironment in HNSCC.

## Methods

### Data source and analysis of differential expressions

Data collection, preparation, and a statement of ethics data on pan-cancer RNA sequencing (RNA-seq) were gathered from the UCSC XENA database (https://xenabrowser.net/datapages/) [26]. The TCGA site was used to download Level 3 RNA RNA-seq expression data and clinical data for HNSCC (502 HNSCC tissues vs. 44 normal neighboring cancer tissues) (https://portal.gdc.cancer.gov/) [21]. Baseline clinical data are shown in Table 1. Transcripts per million reads (TPM) RNA-seq data was log converted (Log2(TPM + 1) before analysis. The gene expression dataset (GSE51985) was obtained based on literature analysis, and it included ten laryngeal squamous cell carcinoma (LSCC) tissue samples and ten neighboring non-tumor tissue samples, and the *CELSR3* expression gene was screened from the dataset [8]. We used TCGA to examine the difference in *CELSR3* expression between HNSCC and normal head and neck tissues. Because the TCGA are open, publicly accessible databases, the data collected from them was following all existing laws, rules, and policies for the protection of human beings, and all subjects participating provided written informed consent.

**Table 1:** Baseline clinical data

Characteristic	Low expression of *CELSR3*	High expression of *CELSR3*	*p*	statistic	method
*n*	251	251			
T stage, *n* (%)			0.052	7.74	Chisq.test
T1	19 (3.9%)	14 (2.9%)			
T2	61 (12.5%)	83 (17%)			
T3	76 (15.6%)	55 (11.3%)			
T4	86 (17.7%)	93 (19.1%)			
N stage, *n* (%)			0.376		Fisher.test
N0	122 (25.4%)	117 (24.4%)			
N1	44 (9.2%)	36 (7.5%)			
N2	68 (14.2%)	86 (17.9%)			
N3	3 (0.6%)	4 (0.8%)			
M stage, *n* (%)			0.214		Fisher.test
M0	233 (48.8%)	239 (50.1%)			
M1	4 (0.8%)	1 (0.2%)			
Clinical stage, *n* (%)			0.058	7.5	Chisq.test
Stage I	12 (2.5%)	7 (1.4%)			
Stage II	44 (9%)	51 (10.5%)			
Stage III	61 (12.5%)	41 (8.4%)			
Stage IV	125 (25.6%)	147 (30.1%)			
Gender, *n* (%)			0.480	0.5	Chisq.test
Female	71 (14.1%)	63 (12.5%)			
Male	180 (35.9%)	188 (37.5%)			
Age, *n* (%)			0.055	3.7	Chisq.test
≤ 60	111 (22.2%)	134 (26.7%)			
> 60	139 (27.7%)	117 (23.4%)			
Histologic grade, *n* (%)			0.021		Fisher.test
G1	40 (8.3%)	22 (4.6%)			
G2	153 (31.7%)	147 (30.4%)			
G3	52 (10.8%)	67 (13.9%)			
G4	0 (0%)	2 (0.4%)			
Smoker, *n* (%)			0.005	7.98	Chisq.test
No	41 (8.3%)	70 (14.2%)			
Yes	201 (40.9%)	180 (36.6%)			
Alcohol history, *n* (%)			0.248	1.34	Chisq.test
No	85 (17.3%)	73 (14.9%)			
Yes	159 (32.4%)	174 (35.4%)			
Radiation therapy, *n* (%)			0.421	0.65	Chisq.test
No	81 (18.4%)	73 (16.6%)			
Yes	138 (31.3%)	149 (33.8%)			
Age, median (IQR)	61.5 (54, 69)	60 (53, 68.5)	0.248	33,244.5	Wilcoxon

### Exploring the association of survival with CELSR3 in HNSCC

The receiver operating characteristic (ROC) curve was built using the pROC of the R package to assess the diagnostic value of *CELSR3* expression level, and the area under the curve (AUC) under a 95% confidence interval (CI) was determined to determine the diagnostic efficacy. Following that, we ran univariate and multivariate Cox regression analyses to determine the best terms for building nomograms. Using the “forestplot” R tool and R software, a forest was used to display the *p*-value, HR, and 95% CI for each variable. To forecast the overall recurrence rate in 1, 3, and 5 years, a nomogram was built based on the results of the multivariate Cox proportional hazards analysis. The R program “rms” was used to calculate the likelihood of recurrence for individual patients based on the points assigned to each risk factor. In TCGA, we extracted survival data for each sample. *CELSR3* and the prognosis of HNSCC patients were studied using indicators such as overall survival (OS), disease-specific survival (DSS), and progression-free interval (PFI). The survival analysis of HNSCC was performed using the Kaplan–Meier (KM) and log-rank tests (*p* < 0.05), and survival curves were examined using the “survminer” and “survivor” R packages.

### GEPIA analysis

The Gene Expression Profiling Interactive Analysis (GEPIA) web server is a novel interactive web server. Its website is located at http://gepia.cancer-pku.cn/index.html [22]. This database offers the online analysis of data from the TCGA and GTeX studies. In our investigation, we used the TCGA expression dataset to compare *CELSR3* levels in HNSCC with normal head and neck tissues. Furthermore, we used GEPIA to assess the prognostic significance of *CELSR3* mRNA expression in HNSCC and create a survival curve with a log-rank p-value.

### Immune infiltration analysis

Individual immune cell types, such as T helper cells, Tcm cells, Th2 cells, and others, were assessed using single-sample GSEA (ssGSEA). The analysis was carried out with the help of the gene set variation analysis (GSVA) software [2, 9]. The TIMER database (https://cistrome.shinyapps.io/timer/) was used to investigate the relationships between HNSCC and TIICs [15]. GSVA package of R was used to analyze the correlations between *CELSR3* expression levels and the infiltrations of B cells, natural killer (NK) cells, the cluster of differentiation 4 T cells (CD4 T cells), cluster of differentiation 8 T cells (CD8 T cells), regulatory T cell (Treg cells), Macrophage M1 cell and T cell follicular helper.

### Immune-checkpoint analysis

Sialic acids binding Ig like lectin 15 (*SIGLEC15*), T cell immunoreceptor with Ig and ITIM domains (*TIGIT*), cytotoxic T lymphocyte-associated antigen-4 (*CTLA4*), the cluster of differentiation 274 (*CD274*), hepatitis A virus cellular receptor 2 (*HAVCR2*), lymphocyte-activation gene 3 (*LAG3*), programmed cell death 1 (*PDCD1*), and programmed cell death 1 ligand 2 (*PDCD1LG2*) were selected to be immune-checkpoint–relevant transcripts and the expression data of these eight genes were extracted. The expression of the immune checkpoints and the co-expression of *CELSR3* with these immune checkpoints were evaluated using the R packages “ggplot2,” “pheatmap,” and “immuneeconv.” Using the TIDE method, potential immune checkpoint blockade responses were anticipated [10].

### LinkedOmics analysis

LinkedOmics is a public website at http://www.linkedomics.org/login.php that provides 32 TCGA cancer types of numerous omics data, including the Clinical Proteomics Tumor Analysis Consortium federation. We used this online tool to discover and analyze *CELSR3* co-expression genes in a TCGA head and neck cancer cohort of 502 samples. The Linkfinder data has been signed and sorted. The gene set enrichment analysis (GSEA) approach is then used to undertake GO analyses such as molecular function (MF), biological process (BP), cellular component (CC), and KEGG analysis.

### Statistical analysis

The log2-transformation was used to normalize gene expression data. R program (version 4.0.2) was used for statistical analysis. *CELSR3* expression analysis in the TIMER and TCGA databases revealed a *p*-value. The GEPIA study produced a survival curve with a *p*-value. The GEPIA study produced a survival curve with a *p*-value. All survival analyses used the Cox proportional hazards model, KM analyses, and the log-rank test. The correlation between the two variables was examined using Spearman’s test or Pearson’s test. *p* values of less than 0.05 were considered significant. For statistical analysis, R software (version 4.0.2) was utilized.

### Cell experiment

#### Cell culture

Head and neck squamous cell lines C6661 and SUN1 were purchased from the Bo Rui Biomedicine Co., LTD, and human oral keratinocyte (HOK) was purchased from the Cell Bank of the Chinese Academy of Sciences (Shanghai). The above cells were cultured in high-glucose DMEM medium with 10% fetal bovine serum, 100 U/mL penicillin and 100 U/mL streptomycin, and placed in an incubator containing 5% CO2 at 37 °C. All cells were passaged 3–4 times before use.

#### RNA extraction and RT-qPCR

Total RNA was extracted from HNSCC tissues and cells by TRIzol method, and the RNA was reversed into corresponding cDNA by reverse transcription kit. RT-qPCR was performed using cDNA as template. The primers were PRMT5 justice chain: 5ʹ-CTGTCTTCCATCCGCGTTTCA-3ʹ, antisense chain: 5ʹ-CTGTCTTCCATCCGCGTTTCA-3ʹ; GAPDH justice chain: 5ʹ-CATCTCTGCCCCCTCTGCTGA-3ʹ, antisense chain: 5ʹ-GGATGACCTTGCCCACAGCCT-3ʹ.

#### Cell transfection

C6661 and SUN1 cells were inoculated into 6-well plates, OE-NC, OE-*CELSR3*-#1 and OE-*CELSR3*-#2 were transfected with Lipofectamine 3000 reagent, and fresh medium was replaced after transfection for 24–48 h.

#### The CCK-8 experiment

The transfected C6661 and SUN1 cells were inoculated into 96-well plates with 2 × 103 cells per well, and 100 μL medium containing CCK-8 was added to each well at a ratio of 1:9, and incubated at 37 °C for 1 h without light. Cell proliferation was detected at different time points (0, 24, 48, 72, 96 H). At the wavelength of 450 nm, the corresponding OD value was detected by enzyme-labeled instrument, and the cell proliferation curve was drawn.

## Results

### CELSR3 expression in pan-cancer and HNSCC patients

**Fig. 1:**
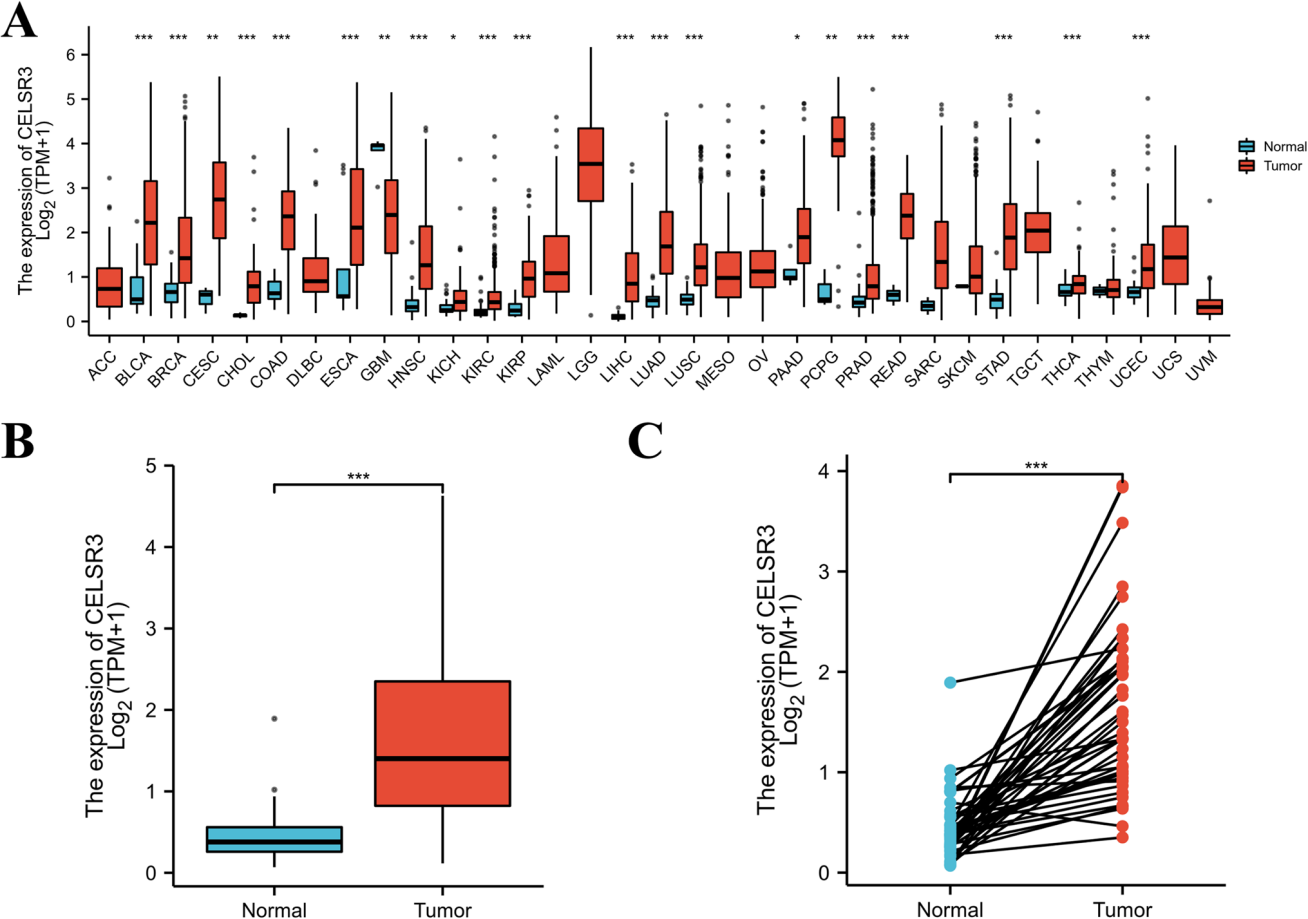
*CELSR3* expression in pan-cancer and HNSCC patients. **A** *CELSR3* expression levels in different tumor types. **B** *CELSR3* expression is significantly higher in tumor tissues than in normal tissues. ***p < 0.001. **C** *CELSR3* expression in 43 HNSCC tissues and their paired adjacent para-carcinomatous tissues.*p < 0.05

Using the data from TCGA, we found that *CELSR3* expression was significantly elevated in 21 of 33 cancer types, including bladder urothelial carcinoma (BLCA), bladder urothelial carcinoma (BRCA), cholangiocarcinoma (CHOL), colon adenocarcinoma (COAD), kidney renal esophageal carcinoma (ESCA), kidney renal clear cell carcinoma (KIRC), among others (Fig. 1A). *CELSR3* expression was also significantly higher in HNSCC tissues compared to normal samples (Fig. 1B). *CELSR3* expression was similarly higher in cancer tissues than in matched normal neighboring tissue samples in the 43 pared samples (Fig. 1C).

### The expression of CELSR3 is associated with patients’ survival in HNSCC

**Fig. 2:**
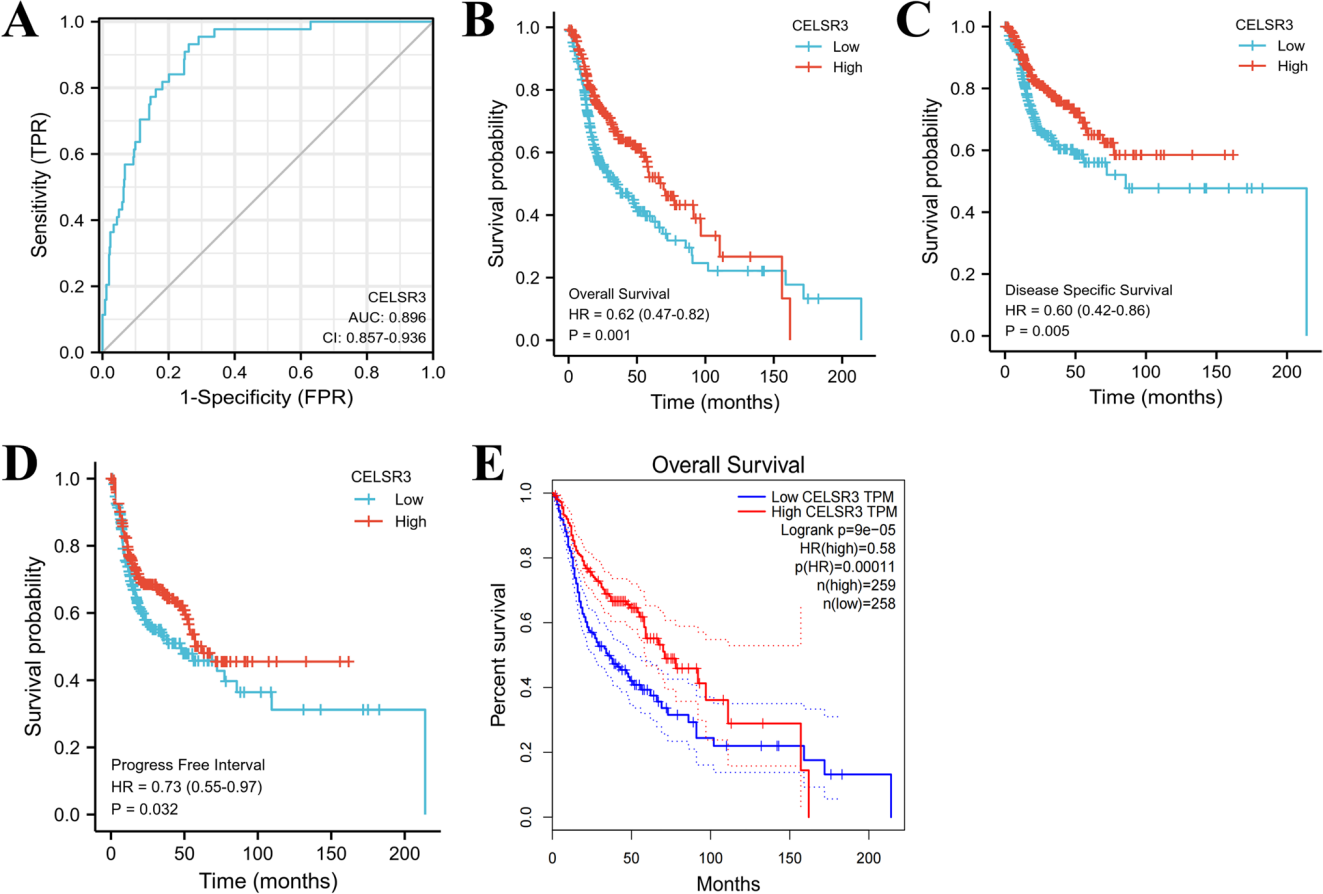
*CELSR3* expression level has potential diagnostic and prognostic values for patients with HNSCC. **A** The diagnostic value of *CELSR3* expression was evaluated using the ROC curve. **B** Kaplan–Meier (K–M) survival curves for OS. **C** K–M survival curves for DSS. **D** K–M survival curves for PFI. **E** K–M survival curves for OS constructed from GEPIA

*CELSR3* expression survival data in HNSCC patients were examined. A ROC curve was created to assess the diagnostic utility of the *CELSR3* expression level. *CELSR3* expression has a high sensitivity and specificity for HNSCC diagnosis, as demonstrated in Fig. 2A. The AUC (area under the curve) was 0.896. Following that, the prognostic value of *CELSR3* expression was assessed using a KM analysis. The median was chosen as the cut-off value for the high and low *CELSR3* expression groups. The results showed that patients with higher *CELSR3* mRNA expression had longer OS (*p* < 0.001) (Fig. 2B), DSS (*p* < 0.005) (Fig. 2C), PFI (*p* < 0.05) (Fig. 2D). As a result, *CELSR3* mRNA expression in HNSCC is linked to survival. The result of OS from GEPIA is also consistent with our results (Fig. 2E).

### Prognostic potential of CELSR3 in HNSCC

**Fig. 3:**
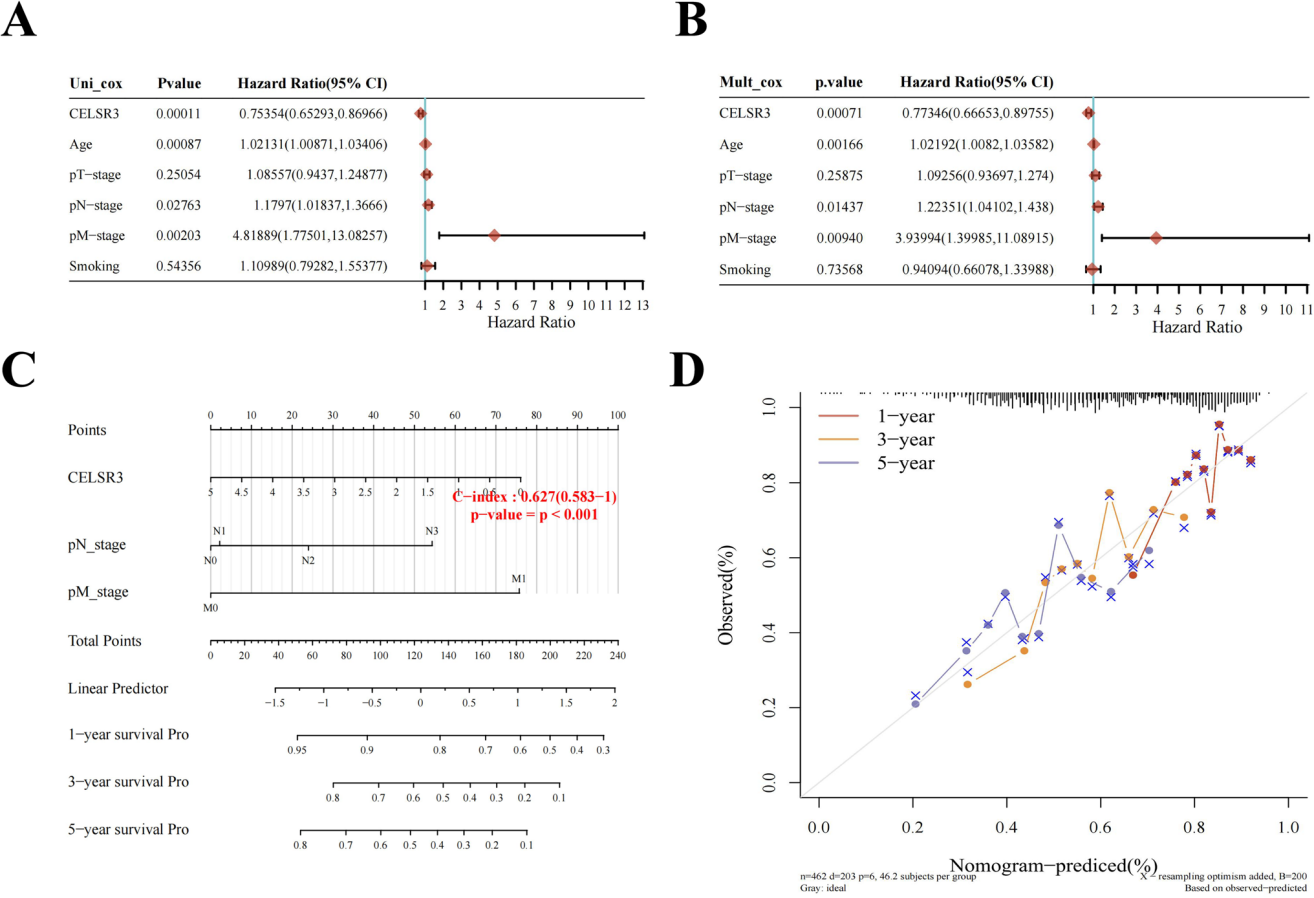
Prognostic efficacy of *CELSR3* expression levels in patients with HNSCC. **A** Results of univariate Cox regression analyses displayed using forest plots. **B** Results of multivariate Cox regression analyses displayed using forest plots. **C** Nomogram for predicting clinical outcomes associated with *CELSR3* expression in HNSCC patients. **D** Calibration plots validating 1-, 3-, and 5-year clinical outcomes for HNSCC patients

Then, using a variety of clinical features and *CELSR3* expression levels, we ran univariate and multivariate Cox regression analyses. Furthermore, univariate and multivariate Cox regression analyses illustrated that *CELSR3* expression (*p* < 0.01), pN-stage (*p* < 0.05), and pM-stage (*p* < 0.01) were important independent factors to the prognosis of HNSCC (Fig. 3A, B). We then created a nomogram that comprised only three independent prognostic markers (*CELSR3*, pN-stage, and pM-stage) to offer doctors a quantitative guideline for predicting the probability of 1-, 3-, and 5-year OS in HNSCC patients (Fig. 3C). Each patient is assigned a total score by summing each prognostic parameter point, with a larger total score indicating a worse outcome. Furthermore, the calibration curves revealed that the nomogram did well in estimating 1-, 3-, and 5-year OS (Fig. 3D). As a result, *CELSR3* may be a possible HNSCC diagnostic marker.

### Association between CELSR3 expression and immune cell infiltration and immune molecule expression levels

**Fig. 4:**
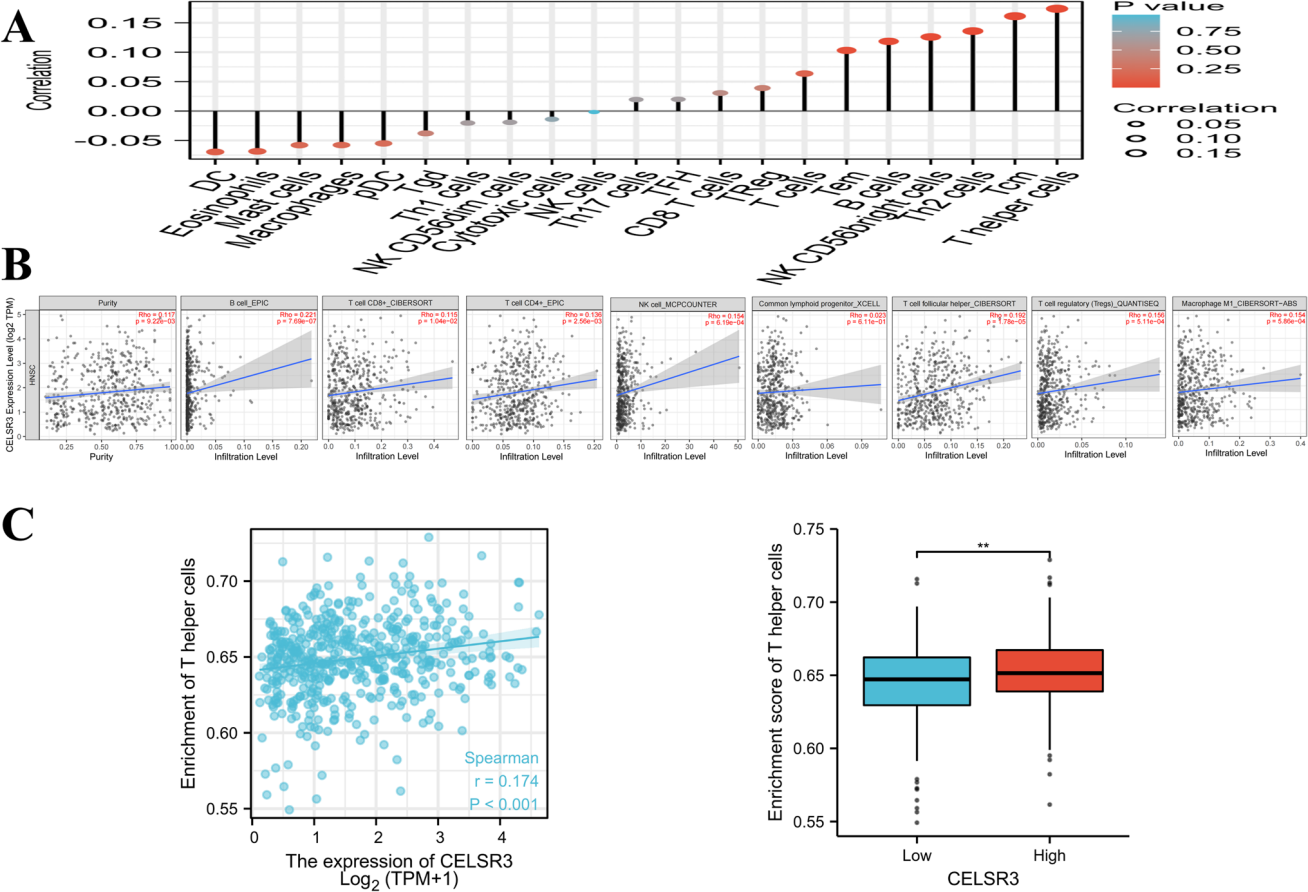
Relationship between *CELSR3* expression and immune cell infiltration in the tumor microenvironment. **A** Correlations between *CELSR3* expression and 21 types of immune cell infiltrations. **B** *CELSR3* expression was positively correlated with the infiltration of B cells, NK cells, T cell follicular helper cells, CD4+ T cells, CD8+ T cells, T cell follicular helper cells, common lymphoid progenitor, and Macrophage M1 cells. **C** The level of T helper cell infiltration in the high-expression group of *CELSR3* was substantially different from that in the low-expression group (*p* < 0.001)

We used Spearman’s correlation to reveal a link between *CELSR3* expression levels and immune cell infiltration levels assessed by ssGSEA in the HCC tumor microenvironment to better understand the effect of *CELSR3* expression on the tumor microenvironment. T helper cells were significantly positively correlated with *CELSR3* expression (Spearman *R* = 0.174, *p* < 0.001). Other immune cell subsets, such as Th2 cells, B cells, effective memory T cells (Tem cells), and others, are likewise related to *DNTTIP1* expression (Fig. 4A). Following that, Using the CIBERSORT algorithm, we discovered that *CELSR3* expression was positively correlated with the infiltration of B cells, NK cells, T cell follicular helper cells, cluster of differentiation 4-positive T cells (CD4+ T cells), cluster of differentiation 8-positive T cells (CD8+ T cells), T cell follicular helper cells, common lymphoid progenitor, and Macrophage M1 cells (Fig. 4B). *CELSR3* expression levels were significantly positively linked with immune cell infiltrations (*p* < 0.001). The level of T helper cell infiltration in the high-expression group of *CELSR3* was substantially different from that in the low-expression group (*p* < 0.001) (Fig. 4C).

**Fig. 5:**
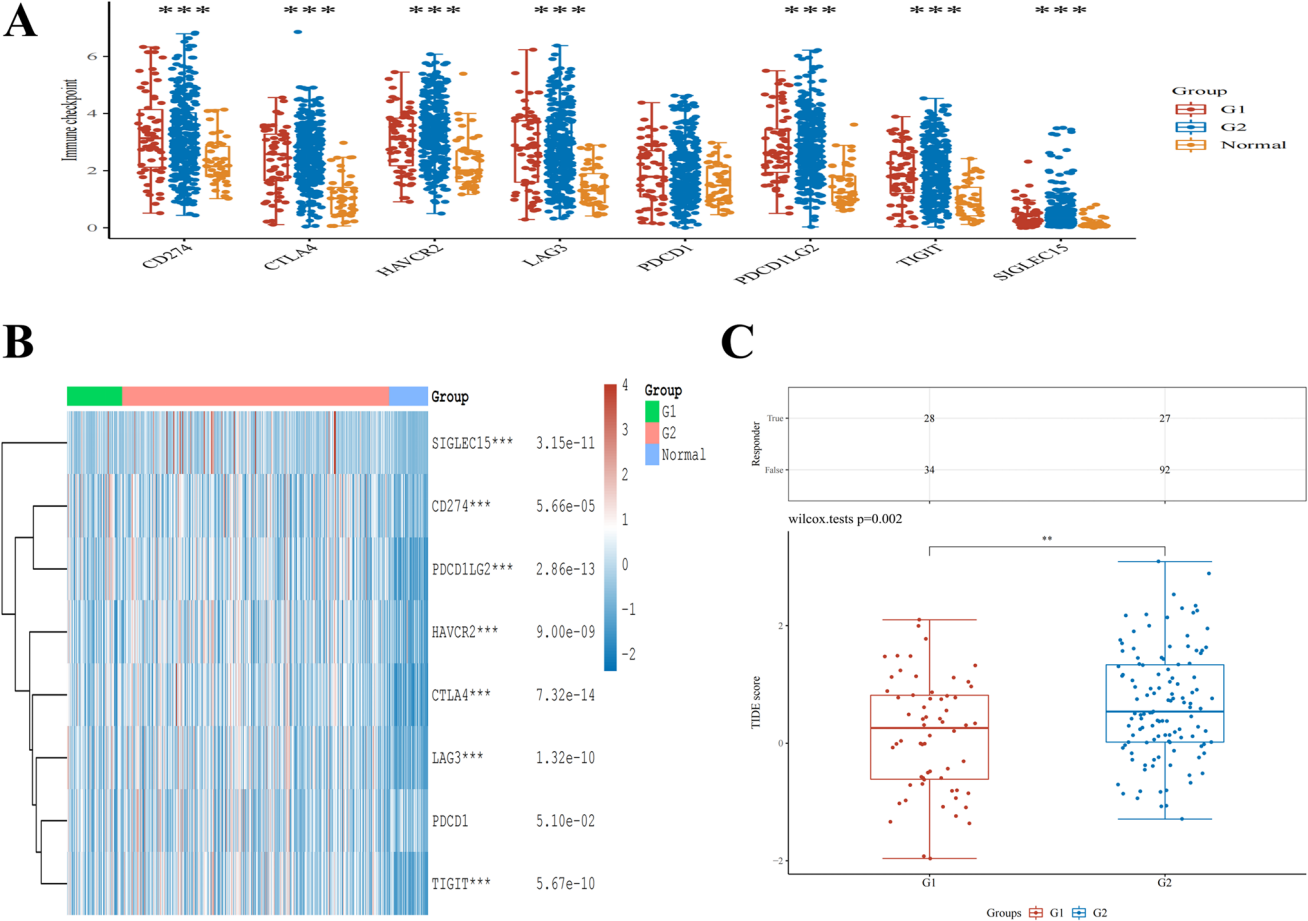
The expression of immune-checkpoints in HNSCC. **A**, **B** Different expressions of immune checkpoints in grade I and II of HNSCC. **C** Different responses to immune checkpoint blockade in grade I and II of HNSCC

Similarly, we investigated the relationship between *CELSR3* and immunoassays, as well as the expression of immune checkpoints, such as *CD274, CTLA4, HAVCR2, LAG3, PDCD1, PDCD1LG2, TIGIT,* and *SIGLEC15* (Figs. 5A), in WHO grade I and II HNSCC. The results illustrated that *LAG3* (*p* < 1.32e−10), *CTLA4* (*p* < 7.32e−14), *CD274* (*p* < 5.66e−05), *HAVCR2* (*p* < 9.00e−09), *TIGIT* (*p* < 5.67e−10), and *SIGLEC15* (*p* < 3.15e−11) were upregulated in WHO grade II compared with WHO grade I of HNSCC (Fig. 5B). Furthermore, we discovered that HNSCC patients in WHO grade II responded better to immune checkpoint blockade than HNSCC patients in WHO grade I (Fig. 5C). As a result, *CELSR3* could be a viable immunotherapy target.

### Enrichment analysis of CELSR3 neighborhood genes in HNSCC

**Fig. 6:**
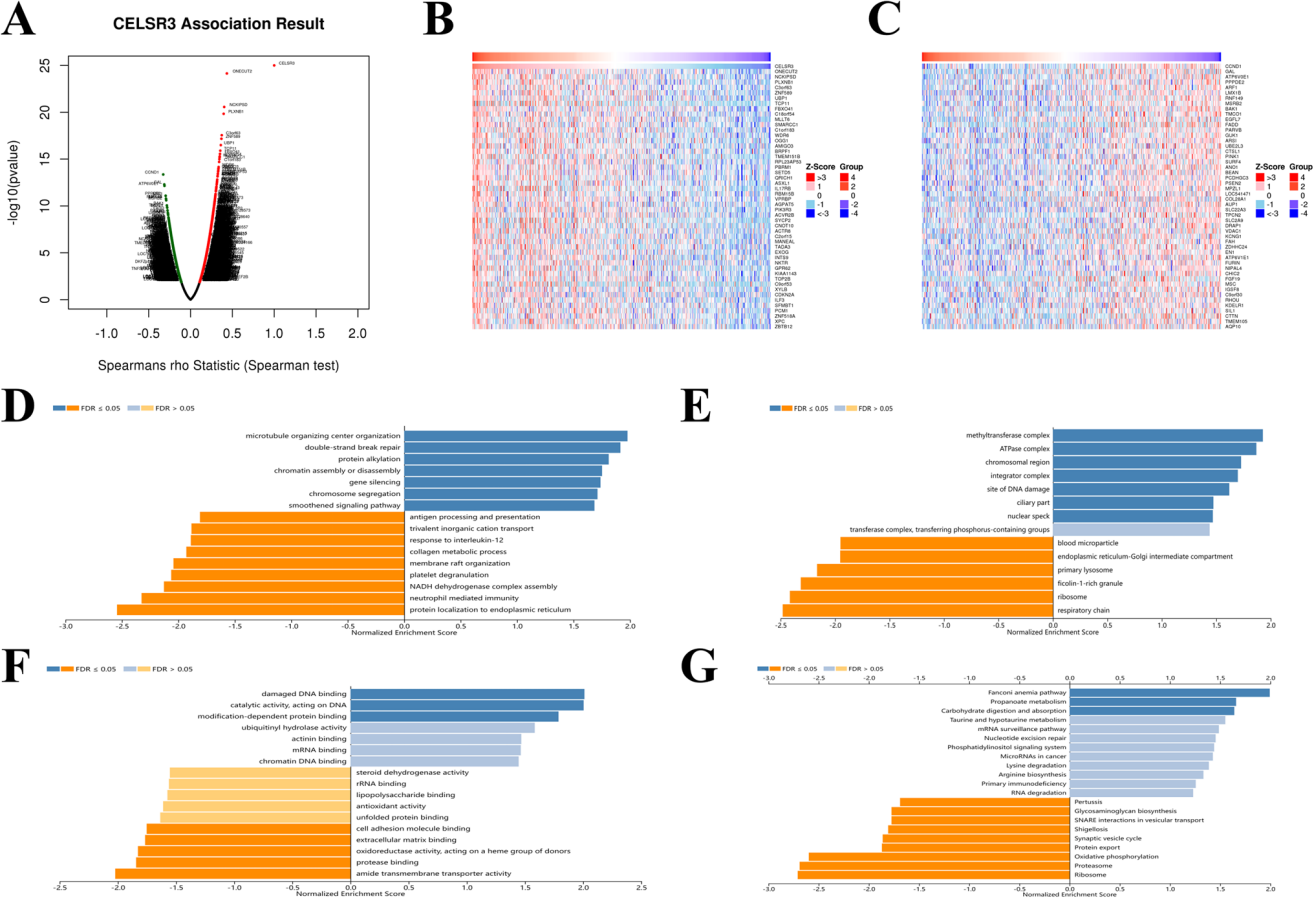
Genes differentially expressed in association with *CELSR3* in HNSCC, and enrichment analysis of the genes altered in the *CELSR3* neighborhood in HNSCC. **A** Volcano plot. **B** Heat maps of the top 50 genes positively correlated with *CELSR3*. **C** Heat maps of the top 50 genes negatively correlated with *CELSR3*. **D** Biological processes. **E** Cellular components. **F** Molecular functions. **G** KEGG pathway analysis

To investigate the functional network of *CELSR3* neighborhood genes in HNSCC, we first used LinkedOmics to identify *CELSR3* neighborhood genes. The outcomes are depicted in a volcano plot (Fig. 6A). In addition, the heat map displayed the top 50 negatively and positively significantly linked genes (Figs. 6B, C). GSEA significant GO term analysis revealed that genes correlated with *CELSR3* were primarily found in methyltransferase complexes, ATPase complexes, chromosomal regions, and integrator complexes, where they were involved in microtubule organizing center organization, double-strand break repair, protein alkylation, and chromatin assembly or disassembly. These linked genes also performed the functions of damaged DNA binding, catalytic activity, acting on DNA, modification-dependent protein binding, and ubiquitinyl hydrolase activity (Figs. 6D–F). KEGG pathway analysis revealed that these *CELSR3*-related genes were primarily prominent in signal pathways such as Fanconi anemia, Propanoate metabolism, Carbohydrate digestion and absorption, and Taurine and hypotaurine metabolism (Fig. 6G).

### Overexpression efficiency of CELSR3 in HNSCC cells

**Fig. 7:**
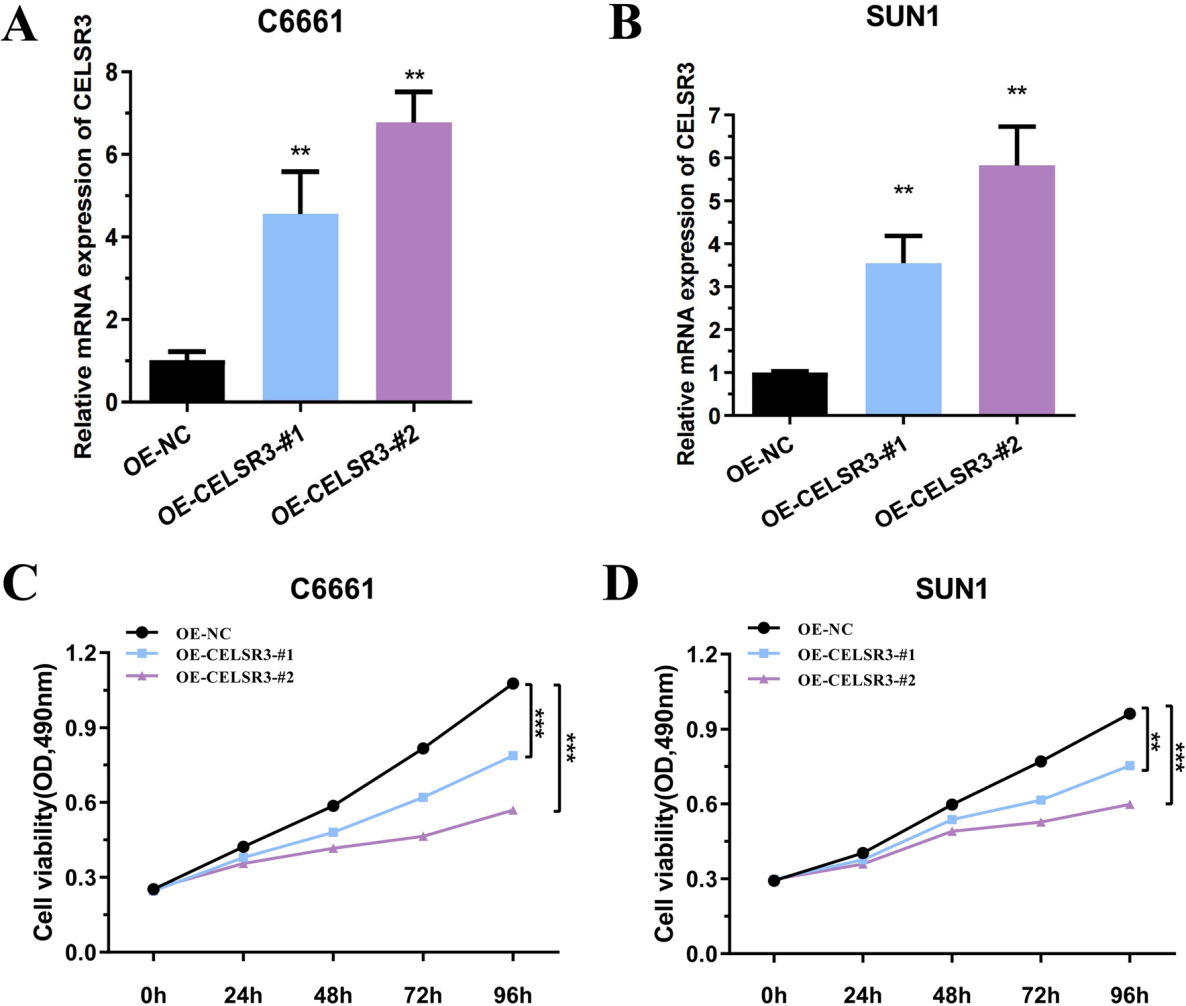
Overexpression efficiency of *CELSR3* in C6661 and SUN1 cells. CCK-8 assay was used to detect the effect of *CELSR3* on the proliferation of head and neck squamous cell cells. A, **B** OE-*CELSR3* significantly up-regulated the expression of *CELSR3* in C6661 and SUN1 cells. **C**, **D** The proliferation capacity of C6661 and SUN1 cells in OE-*CELSR3*-#1 and OE-*CELSR3*-#2 groups was significantly down-regulated (*p* < 0.05)

RT-qPCR verified the overexpression efficiency of *CELSR3* in C6661 and SUN1 cells, and the results showed that both OE-*CELSR3* could significantly up-regulate *CELSR3* expression. It was demonstrated that OE-*CELSR3* significantly up-regulated the expression of *CELSR3* in C6661 and SUN1 cells (Fig. 7A, B).

### Overexpression of CELSR3 inhibited the proliferation of HNSCC cells

The effect of *CELSR3* on the proliferation ability of HNSCC cells was detected by CCK-8 assay. Compared with the control group (NC group), the proliferation capacity of C6661 and SUN1 cells in OE-*CELSR3*-#1 and OE-*CELSR3*-#2 groups was significantly down-regulated (*p* < 0.05) (Fig. 7C, D).

## Discussion

HNSCC is the world’s sixth most common cancer. It comes from the oral cavity, nasal cavity, nasopharynx, larynx, hypopharynx, and oropharynx, among other places in the head and neck. 90% of head and neck cancers are squamous cell carcinomas (SCC) [31]. Because of the delayed early identification and high recurrence risk of HNSCC, approximately 50% of all patients will eventually have a local or regional recurrence. Despite advancements in treatment, the 5-year survival rate remains very low, and some patients continue to have poor treatment effects and poor prognoses [19]. As a result, it is critical to investigate new genes associated with the onset and progression of HNSCC, as well as to identify indicators with more specificity and sensitivity, particularly for additional molecular mechanism targets such as immune infiltration, to improve HNSCC treatment. The purpose of this study was to look at the diagnostic significance of the *CELSR3* gene in HNSCC as well as its effect on tumor immune invasion.

The *CELSR3* gene is a member of the flamingo subfamily and the Cadherin superfamily [6]. *CELSR3* may be crucial for cell-to-cell communication throughout the development of the nervous system [23]. According to reports, the *CELSR3* gene may contribute to the development of tumors by regulating neurite-dependent neurite outgrowth and being overexpressed in several human malignancies. Several human cancers, including brain tumors, ovarian cancer, pancreatic cancer, liver cancer, colorectal cancer, and cervical cancer, have been shown to have overexpressed *CELSR3* genes [1, 5, 7, 13]. Finally, we can conclude that *CELSR3* may be involved in tumor formation and development by influencing cell proliferation via the cellular microenvironment.

*CELSR3* expression was discovered to be differentially expressed in numerous forms of cancer and their matching normal tissues in our investigation, including BLCA, BRCA, CHOL, COAD, ESCA, KIRC, and others. Some studies, for example, found that *CELSR3* was highly expressed in the early stages of cancer and remained present throughout the entire cancer process, implying that *CELSR3* may play a key role in the carcinogenesis of hepatocellular carcinoma. *CELSR3* expression in HNSCC was confirmed further, and the results revealed that patients with high *CELSR3* expression had a better prognosis. Furthermore, we discovered that *CELSR3* gene expression was related to pN-stage, and pM-stage. Furthermore, univariate and multivariate Cox regression analysis revealed that *CELSR3* expression was a prognostic factor for HNSCC. As a result, *CELSR3* may have diagnostic significance in HNSCC.

Based on GO and KEGG analyses, we observed that the functional network of *CELSR3* in HNSCC was related to methyltransferase complex, ATPase complex, chromosomal region, integrator complex, where they were involved in microtubule organizing center organization, double-strand break repair, protein alkylation, chromatin assembly or disassembly. These related genes also served as damaged DNA binding, catalytic activity, acting on DNA, modification-dependent protein binding, ubiquitinyl hydrolase activity. KEGG pathway analysis demonstrated that these genes related to *CELSR3* were mainly enriched in signal pathways such as Fanconi anemia pathway, Propanoate metabolism, Carbohydrate digestion and absorption, Taurine and hypotaurine metabolism.

We used Spearman’s correlation to reveal a link between *CELSR3* expression levels and immune cell infiltration levels assessed by ssGSEA in the HCC tumor microenvironment to investigate the effect of *CELSR3* expression on the tumor microenvironment. *CELSR3* expression was highly linked with T helper cells. Other immune cells subsets, such as Th2 cells, B cells, and T cells, are similarly linked to *DNTTIP1* expression. The level of T helper cell infiltration in the *CELSR3* high-expression group was considerably higher than in the low-expression group. *CELSR3* expression was found to be positively connected with B cells, NK cells, T cell follicular helper cells, CD4+ T cells, CD8+ T cells, T cell follicular helper cells, and Macrophage M1 cell infiltration. *CELSR3* expression levels were shown to be substantially linked with immune cell infiltrations. Most cancers boost inhibitory ligand levels by affecting T cell function to avoid immune responses that lead to tumor progression. One of the potential pathways boosting tumor development is hypothesized to be this. Patients with higher T-cell infiltration frequently have a better prognosis [18, 25]. Similarly, we looked at the relationship between *CELSR3* and immunoassays and discovered that *LAG3, CTLA4, CD274, HAVCR2, TIGIT,* and *SIGLEC15* were increased in WHO grade II HNSCC compared to WHO grade I HNSCC. Furthermore, we discovered that HNSCC patients in WHO grade II responded better to immune checkpoint blockade than HNSCC patients in WHO grade I. As a result, *CELSR3* could be a viable immunotherapy target.

A great number of studies have established that tumor immune cell infiltration can alter the efficacy and prognosis of cancer patients receiving chemotherapy, radiation, or immunotherapy [16, 30]. Taken together, *CELSR3*’s influence on immune cell infiltration has a good impact on tumor patients. In HNSCC, *CELSR3* is involved in the recruitment and control of immune-invading cells. Most notably, *CELSR3* expression may influence the immunological microenvironment, which in turn influences the prognosis of HNSCC. The drawback of our study is that a large number of samples were required to verify our findings, and additional clinical samples will be gathered in the future to enhance the data. Furthermore, the underlying immune mechanisms should be investigated, and *CELSR3* should be tested as a biomarker to predict the immune response rate in real-world HNSCC patients.

RT-qPCR verified the overexpression efficiency of *CELSR3* in C6661 and SUN1 cells, and the results showed that both OE-*CELSR3* could significantly up-regulate *CELSR3* expression. It was demonstrated that OE-*CELSR3* significantly up-regulated the expression of *CELSR3* in C6661 and SUN1 cells. The effect of *CELSR3* on the proliferation ability of HNSCC cells was detected by CCK-8 assay. Compared with the control group (NC group), the proliferation capacity of C6661 and SUN1 cells in OE-*CELSR3*-#1 and OE-*CELSR3*-#2 groups was significantly down-regulated.

The article discussed the diagnostic value of *CELSR3* in HNSCC and its effect on tumor immune invasion, which have an important impact on the clinical management of HNSCC patients. Numerous clinical trials are underway to further refine the application of immunotherapy and develop new immunotherapy approaches. However, there is an urgent need for reliable predictive biomarkers for personalized clinical management and new treatment strategies [3]. Chen X et al. suggested that CELSR3 may play an important role in the progression of prostate cancer [4]. Ouyang et al. [20] demonstrated that CELSR3 was highly expressed in the early stage of cancer and was present throughout the entire cancer process, which suggested that CELSR3 may serve a key role in the carcinogenesis of hepatocellular carcinoma. We found that CELSR3 expression is elevated in HNSCC tumor tissues, and high CELSR3 expression is associated with well prognosis, which inhibited the proliferation of NHSCC cells. CELSR3 has the potential to influence tumor formation by controlling tumor-infiltrating cells in the TME. As a result, CELSR3 may have diagnostic significance in HNSCC. In clinical medicine, the application of bioinformatics can not only help doctors to diagnose and treat diseases, but also accelerate the development of new drugs and the implementation of personalized medicine. These findings could help doctors make earlier diagnoses, provide more precise treatments, and provide targets for the development of new drugs. For example, by sequencing the genomes of patients with HNSCC, it may be found that mutations in the CELSR3 are associated with the development of HNSCC. Based on the genomic information of patients with HNSCC, doctors can select the most appropriate drug and dose for the patient, improve treatment effectiveness, and reduce adverse reactions during treatment, and enabling early cancer screening and diagnosis.

## Conclusions

In conclusion, our findings show that *CELSR3* expression is enhanced in HNSCC tumor tissues, and that high *CELSR3* expression is related to well prognosis. *CELSR3* overexpression significantly inhibited the proliferation of HNSCC cells. *CELSR3* has the potential to influence tumor formation by controlling tumor-infiltrating cells in the tumor microenvironment (TME). *CELSR3* could be used as an immunotherapy target.

## Data Availability

The data that support the findings of this study are available from the corresponding author, upon reasonable request.

## Abbreviations

AUC: Area under the curve
BLCA: Bladder urothelial carcinoma
BP: Biological process
BRCA: Breast invasive carcinoma
CC: Cellular component
CD274: Cluster of differentiation 274
CD4 T cells: Cluster of differentiation 4T cells
CD4+ T cells: Cluster of differentiation 4-positive T cells
CD8 T cells: Cluster of differentiation 8T cells
CD8+ T cells: Cluster of differentiation 8-positive T cells
CD8 T cells: Cluster of differentiation 8T cells
*CELSR*: Cadherin EGF LAG seven-pass G-Type
*CELSR3*: Cadherin EGF LAG seven-pass G-Type receptor 3
CHOL: Cholangiocarcinoma
CI: Confidence interval
COAD: Colon adenocarcinoma
CTLA4: Cytotoxic T lymphocyte-associated antigen-4
DNTTIP1: Deoxynucleotidyltransferase terminal interacting protein 1
DSS: Disease-specific survival
ESCA: Esophageal carcinoma
GEPIA: Gene expression profile interaction analysis
GO: Gene ontology
GSEA: Gene set enrichment analysis
GSVA: Gene set variation analysis
HAVCR2: Hepatitis A virus cellular receptor 2
HNSCC: Head and neck squamous cell carcinoma
ICB: Immune checkpoint blockade
KEGG: Kyoto Encyclopedia of Genes and Genomes
KIRC: Kidney renal clear cell carcinoma
KM: Kaplan–Meier
LAG3: Lymphocyte-activation gene 3
LSCC: Laryngeal squamous cell carcinoma
MF: Molecular function
NK: Natural killer
OS: Overall survival
PDCD1: Programmed cell death 1
PDCD1LG2: Programmed cell death 1 ligand 2
PFI: Progression-free interval
RNA-seq: RNA sequencing
ROC: Receiver operating characteristic
SCC: Squamous cell carcinoma
SIGLEC15: Sialic acid binding Ig-like lectin 15
ssGSEA: Single-sample GSEA
TCGA: The cancer genome atlas
Tcm cells: Central memory T cells
Tem cells: Effective memory T cells
TIGIT: T cell immunoreceptor with Ig and ITIM domains
TIICs: Tumor-infiltrating immune cells
TIMER: Tumor immune estimation resource
TME: Tumor microenvironment
TPM: Transcripts per million reads
TReg: Regulatory T cell
WHO: World health organization

## References

[1] Asad M, Wong MK, Tan TZ, Choolani M, Low J, Mori S, Virshup D, Thiery JP, Huang RY (2014). Fzd7 drives in vitro aggressiveness in stem-a subtype of ovarian cancer via regulation of non-canonical wnt/pcp pathway. Cell Death Dis.

[2] Bindea G, Mlecnik B, Tosolini M, Kirilovsky A, Waldner M, Obenauf AC, Angell H, Fredriksen T, Lafontaine L, Berger A, Bruneval P, Fridman WH, Becker C, Pagès F, Speicher MR, Trajanoski Z, Galon J (2013). Spatiotemporal dynamics of intratumoral immune cells reveal the immune landscape in human cancer. Immunity.

[3] Chen S, Yang Y, He S, Lian M, Wang R, Fang J (2023). Review of biomarkers for response to immunotherapy in hnscc microenvironment. Front Oncol.

[4] Chen X, Ma Q, Liu Y, Li H, Liu Z, Zhang Z, Niu Y, Shang Z (2021). Increased expression of celsr3 indicates a poor prognostic factor for prostate cancer. J Cancer.

[5] Erkan M, Weis N, Pan Z, Schwager C, Samkharadze T, Jiang X, Wirkner U, Giese NA, Ansorge W, Debus J, Huber PE, Friess H, Abdollahi A, Kleeff J (2010). Organ-, inflammation- and cancer specific transcriptional fingerprints of pancreatic and hepatic stellate cells. Mol Cancer.

[6] Goffinet AM, Tissir F (2017). Seven pass cadherins celsr1-3. Semin Cell Dev Biol.

[7] Goryca K, Kulecka M, Paziewska A, Dabrowska M, Grzelak M, Skrzypczak M, Ginalski K, Mroz A, Rutkowski A, Paczkowska K, Mikula M, Ostrowski J (2018). Exome scale map of genetic alterations promoting metastasis in colorectal cancer. BMC Genet.

[8] Guan GF, Zheng Y, Wen LJ, Zhang DJ, Yu DJ, Lu YQ, Zhao Y, Zhang H (2015). Gene expression profiling via bioinformatics analysis reveals biomarkers in laryngeal squamous cell carcinoma. Mol Med Rep.

[9] Hänzelmann S, Castelo R, Guinney J (2013). Gsva: Gene set variation analysis for microarray and rna-seq data. BMC Bioinform.

[10] Jiang P, Gu S, Pan D, Fu J, Sahu A, Hu X, Li Z, Traugh N, Bu X, Li B, Liu J, Freeman GJ, Brown MA, Wucherpfennig KW, Liu XS (2018). Signatures of t cell dysfunction and exclusion predict cancer immunotherapy response. Nat Med.

[11] Johnson DE, Burtness B, Leemans CR, Lui VWY, Bauman JE, Grandis JR (2020). Head and neck squamous cell carcinoma. Nat Rev Dis Primers.

[12] Jung AR, Jung CH, Noh JK, Lee YC, Eun YG (2020). Epithelial-mesenchymal transition gene signature is associated with prognosis and tumor microenvironment in head and neck squamous cell carcinoma. Sci Rep.

[13] Katoh M, Katoh M (2007). Comparative integromics on non-canonical wnt or planar cell polarity signaling molecules: transcriptional mechanism of ptk7 in colorectal cancer and that of sema6a in undifferentiated es cells. Int J Mol Med.

[14] Langenhan T, Aust G, Hamann J (2013). Sticky signaling–adhesion class g protein-coupled receptors take the stage. Sci Signal.

[15] Li T, Fan J, Wang B, Traugh N, Chen Q, Liu JS, Li B, Liu XS (2017). Timer: A web server for comprehensive analysis of tumor-infiltrating immune cells. Cancer Res.

[16] Lyu L, Yao J, Wang M, Zheng Y, Xu P, Wang S, Zhang D, Deng Y, Wu Y, Yang S, Lyu J, Guan F, Dai Z (2020). Overexpressed pseudogene hla-dpb2 promotes tumor immune infiltrates by regulating hla-dpb1 and indicates a better prognosis in breast cancer. Front Oncol.

[17] Miao H, Zeng Q, Xu S, Chen Z (2021). Mir-1-3p/celsr3 participates in regulating malignant phenotypes of lung adenocarcinoma cells. Curr Gene Ther.

[18] Oh DY, Kwek SS, Raju SS, Li T, McCarthy E, Chow E, Aran D, Ilano A, Pai CS, Rancan C, Allaire K, Burra A, Sun Y, Spitzer MH, Mangul S, Porten S, Meng MV, Friedlander TW, Ye CJ, Fong L (2020). Intratumoral cd4(+) t cells mediate anti-tumor cytotoxicity in human bladder cancer. Cell.

[19] Oliva M, Spreafico A, Taberna M, Alemany L, Coburn B, Mesia R, Siu LL (2019). Immune biomarkers of response to immune-checkpoint inhibitors in head and neck squamous cell carcinoma. Ann Oncol.

[20] Ouyang X, Wang Z, Yao L, Zhang G (2020). Elevated celsr3 expression is associated with hepatocarcinogenesis and poor prognosis. Oncol Lett.

[21] Smyth GK, Michaud J, Scott HS (2005). Use of within-array replicate spots for assessing differential expression in microarray experiments. Bioinformatics.

[22] Tang Z, Li C, Kang B, Gao G, Li C, Zhang Z (2017). Gepia: A web server for cancer and normal gene expression profiling and interactive analyses. Nucleic Acids Res.

[23] UniProt Consortium T (2018). Uniprot: The universal protein knowledgebase. Nucleic Acids Res.

[24] Usui T, Shima Y, Shimada Y, Hirano S, Burgess RW, Schwarz TL, Takeichi M, Uemura T (1999). Flamingo, a seven-pass transmembrane cadherin, regulates planar cell polarity under the control of frizzled. Cell.

[25] van der Leun AM, Thommen DS, Schumacher TN (2020). Cd8(+) t cell states in human cancer: Insights from single-cell analysis. Nat Rev Cancer.

[26] Vivian J, Rao AA, Nothaft FA, Ketchum C, Armstrong J, Novak A, Pfeil J, Narkizian J, Deran AD, Musselman-Brown A, Schmidt H, Amstutz P, Craft B, Goldman M, Rosenbloom K, Cline M, O'Connor B, Hanna M, Birger C, Kent WJ, Patterson DA, Joseph AD, Zhu J, Zaranek S, Getz G, Haussler D, Paten B (2017). Toil enables reproducible, open source, big biomedical data analyses. Nat Biotechnol.

[27] von Witzleben A, Wang C, Laban S, Savelyeva N, Ottensmeier CH (2020). Hnscc: tumour antigens and their targeting by immunotherapy. Cells.

[28] Wang G, Zhang M, Cheng M, Wang X, Li K, Chen J, Chen Z, Chen S, Chen J, Xiong G, Xu X, Wang C, Chen D (2021). Tumor microenvironment in head and neck squamous cell carcinoma: Functions and regulatory mechanisms. Cancer Lett.

[29] Wang XJ, Zhang DL, Xu ZG, Ma ML, Wang WB, Li LL, Han XL, Huo Y, Yu X, Sun JP (2014). Understanding cadherin egf lag seven-pass g-type receptors. J Neurochem.

[30] Zhang H, Liu H, Shen Z, Lin C, Wang X, Qin J, Qin X, Xu J, Sun Y (2018). Tumor-infiltrating neutrophils is prognostic and predictive for postoperative adjuvant chemotherapy benefit in patients with gastric cancer. Ann Surg.

[31] Zhou LQ, Hu Y, Xiao HJ (2021). The prognostic significance of survivin expression in patients with hnscc: a systematic review and meta-analysis. BMC Cancer.

